# Small intestinal follicular lymphoma induced by methotrexate: a case report

**DOI:** 10.1186/s12876-021-01849-8

**Published:** 2021-07-08

**Authors:** Yui Osaki, Hideto Kawaratani, Hiroki Kachi, Kyohei Matsuura, Yuki Tsuji, Takahiro Ozutsumi, Hirotetsu Takagi, Masanori Furukawa, Yasuhiko Sawada, Akira Mitoro, Junichi Yamao, Hitoshi Yoshiji

**Affiliations:** The Department of Gastroenterology and Metabolism, Nara Medical University, Kashihara, Nara 634-8522 Japan

**Keywords:** Methotrexate, Ileum, Lymphoproliferative disorder, Follicular lymphoma, Case report

## Abstract

**Background:**

Methotrexate-associated lymphoproliferative disorder (MTX-LPD) is a rare but critical complication that develops in patients treated with MTX. Although MTX-LPD has been recently reported, the incidence of follicular lymphoma in the intestine is very low.

**Case presentation:**

A 73-year-old woman who had been receiving MTX for over 10 years visited our hospital complaining of postprandial abdominal pain and nausea. Upper and lower digestive tract endoscopies did not show any abnormal findings. A patency capsule was stagnated at the proximal part of the ileum with a mild dilation on the oral side. An oral balloon endoscopy revealed shallow ulcerative lesions in the jejunum. She was diagnosed with MTX-LPD based on histopathological findings. The symptoms did not improve with the discontinuation of MTX, and the patient required partial resection of the small intestine. The test result for Epstein-Barr virus-encoded small RNA was negative. She was diagnosed with follicular lymphoma based on the histology findings of a surgical specimen. Postoperative positron emission tomography-computed tomography and bone marrow aspiration did not show any findings of lymphoma. On follow-up, no recurrence was noted four years after the surgery.

**Conclusions:**

Herein, we report the first case of follicular lymphoma that occurred in the small intestine, negative for Epstein-Barr virus-encoded small RNA. If intestinal symptoms occur during MTX administration, it is important to directly observe by endoscopy and perform histological examination.

## Background

The treatment of rheumatoid arthritis (RA) has evolved in recent years. Methotrexate (MTX) is the first-line therapy for RA [1]. The hyperimmune state of RA itself or the immunosuppressive state induced by MTX administration might contribute to the development of lymphoproliferative disorder (LPD). Methotrexate-associated lymphoproliferative disorder (MTX-LPD) was categorized as an “other iatrogenic immunodeficiency-associated LPD,” which is similar to immunodeficiency-associated LPD, including post-transplant LPD and human immunodeficiency-associated LPD, in the 4th edition of the World Health Organization classification of tumors of hematopoietic and lymphoid tissues [2]. In approximately 40–60 % of patients with MTX-LPD, discontinuing MTX results in the resolution of LPD [3]. Approximately 40–50 % of MTX-LPD cases occur in extranodal sites, in the gastrointestinal tract, skin, liver, lung, and kidney [4]. MTX-LPD has various histopathological features. Among patients treated with MTX, diffuse large B-cell lymphoma is the most common feature (35–60 %), followed by classical Hodgkin’s lymphoma (12–25 %); in contrast, follicular lymphoma is very rare (approximately 3 %) [5, 6]. Herein, we report the first case of follicular lymphoma of MTX-LPD in the small intestine that was completely treated by surgery and MTX withdrawal.

## Case presentation

**Fig. 1:**
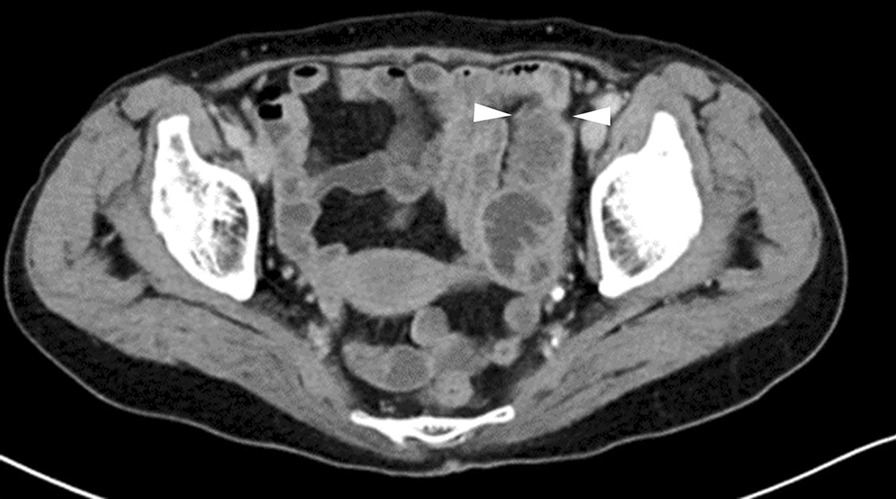
Abdominal computed tomography (CT) showed slight thickening of the small intestinal wall (arrow head)

**Fig. 2:**
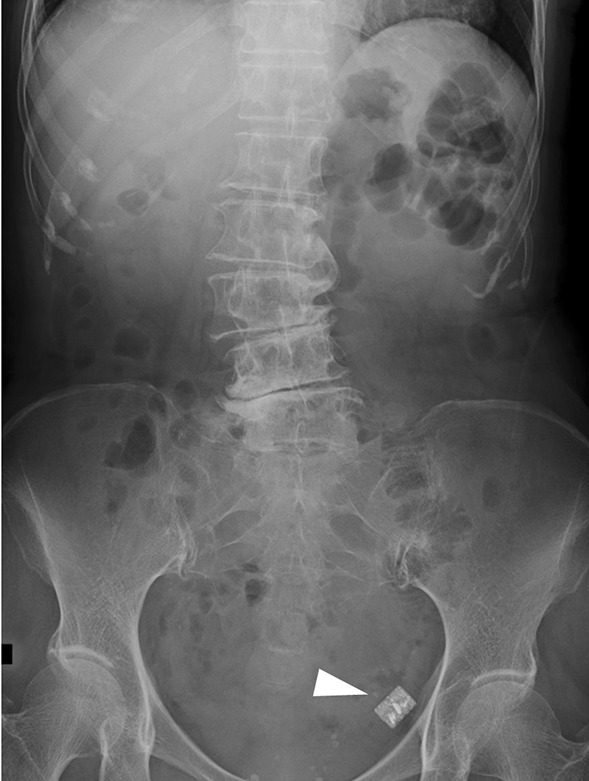
Abdominal X-ray showed the patency capsule (arrow head) remains in the small intestine

**Fig. 3:**
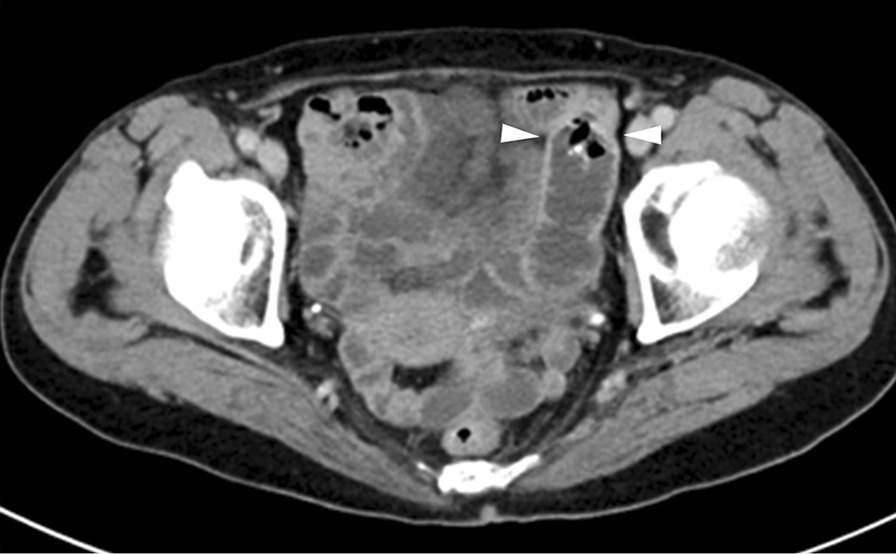
Abdominal CT showed the patency capsule stagnated in the proximal part of the ileum (arrow head) and mild dilatation of the oral part of the small intestine

**Fig. 4:**
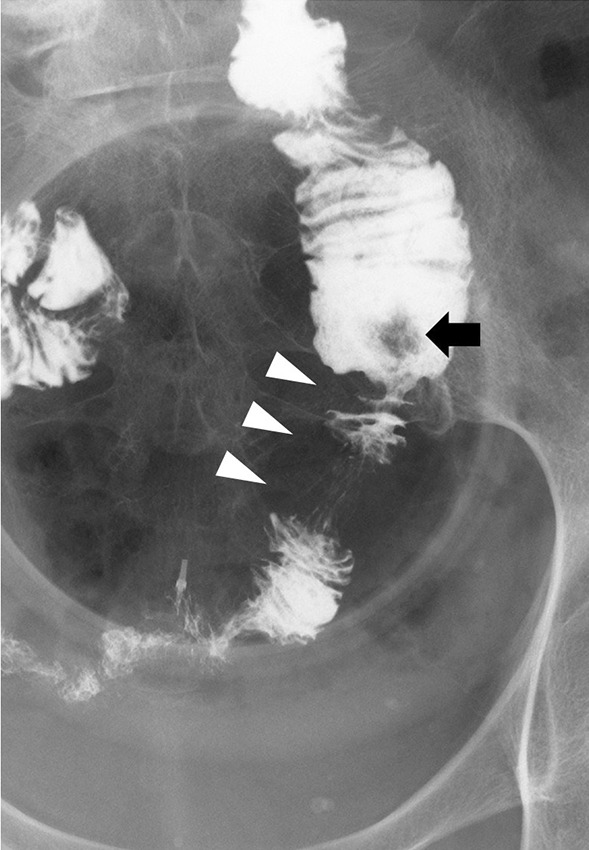
Barium small bowel enema showed a circumferential stenosis at the proximal part of the ileum (arrow head) and stagnated patency capsule (arrow)

**Fig. 5:**
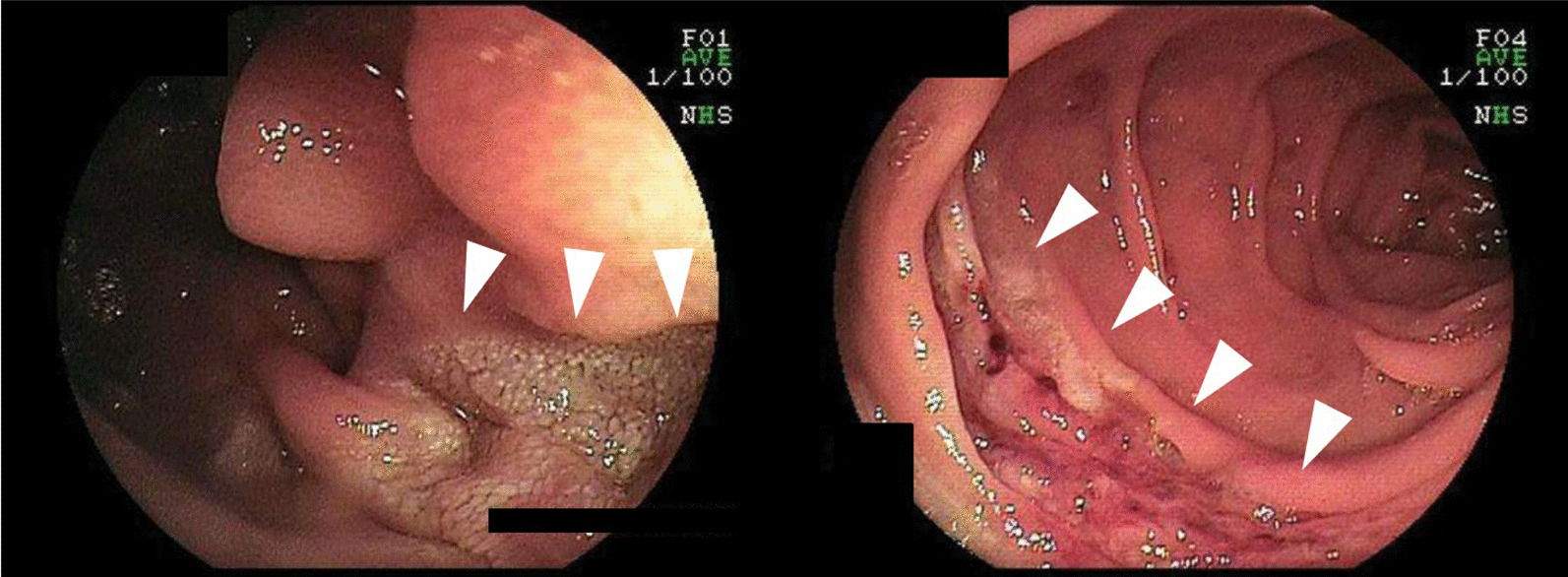
Transoral balloon endoscopy showed several shallow ulcerative lesions (arrow heads)

**Fig. 6:**
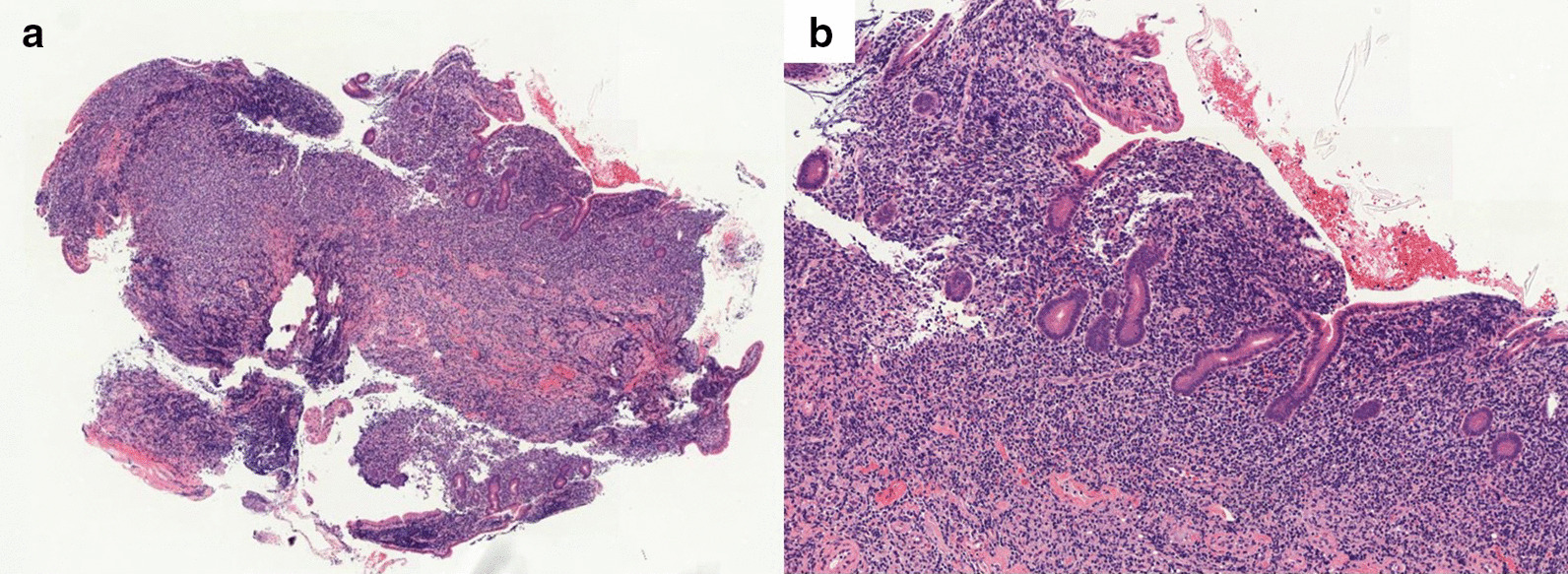
A biopsy from the ulcerative lesion showed densely proliferating lymphocytes in the lamina propria and a marked reduction in gland ducts

**Fig. 7:**
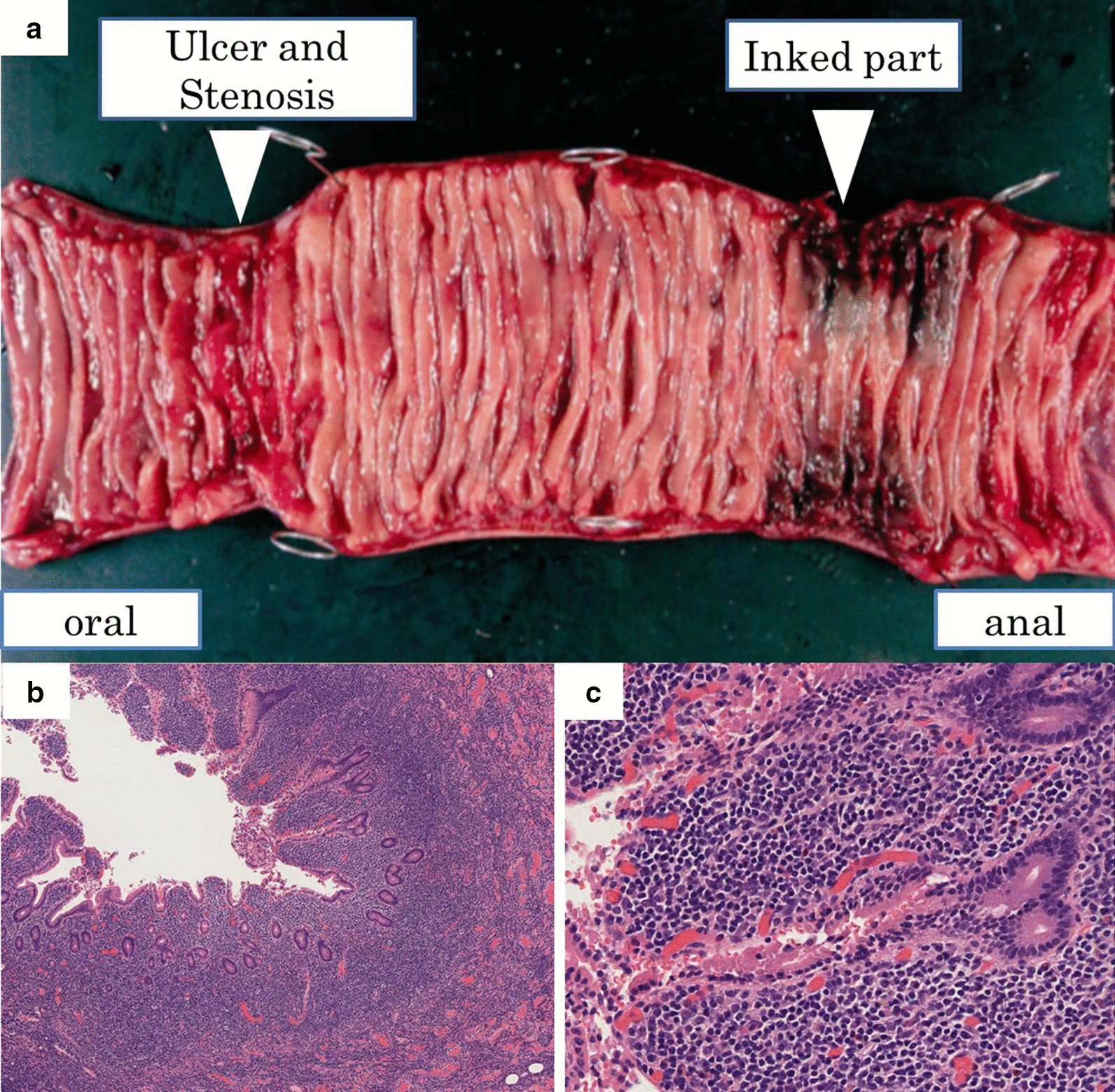
**a** Surgical specimen; circumscribed ulcer and mild stenosis at 3 cm of the oral side from the inked part. Small to medium-sized lymphocytes, which are relatively monotonous, are densely proliferated in the lamina propria with hematoxylin and eosin staining in **b** low-power field (40 ×) and **c** high-power field (400 ×)

A 73-year-old woman who had suffered from RA for 15 years was treated with oral MTX 6 mg/week for over 10 years; the cumulative dose of MTX at the time of presentation to our hospital was approximately 3,000 mg. She had no relevant family history and psycho-social history. She complained of lower abdominal pain, abdominal distention, and nausea after eating in June 2016. She presented to the primary care physician in August 2016 with complaints of exacerbation of lower abdominal pain. Abdominal plain computed tomography (CT), esophagogastroduodenoscopy, and ileocolonoscopy including terminal ileum showed no abnormalities including ulcers. The symptom improved on fasting; however, it recurred when she resumed eating. Therefore, she was referred to our hospital for further investigation. Her general physical condition was good, and no abnormal findings were observed. Her height and body weight were 155.0 cm and 53.2 kg, respectively, and she experienced no body weight loss. Her axillary temperature was 35.9° C, and her blood pressure was 122/72 mmHg. There were no palpable swollen lymph nodes on physical examination. Her abdominal findings were unremarkable, with no hepatosplenomegaly. Laboratory findings were as follows: white blood cell count, 4.3 × 10^3^/µL with normal differential count; hemoglobin, 13.0 g/dL; platelet count, 16.4 × 10^4^/L; total protein, 6.9 g/dL; albumin, 4.2 g/dL; lactate dehydrogenase, 173 U/L; C-reactive protein, 0.1 mg/dL; aspartate aminotransferase, 60 IU/L; alanine transaminase, 23 IU/L; alkaline phosphatase, 207 IU/L; carcinoembryonic antigen, 2.6 ng/mL; CA19-9, 1.0 ng/mL; IgA, 365.3 mg/dL; IgG, 1382.7 mg/dL; IgM, 48.4 mg/dL; antinuclear antibody, negative; rheumatoid factor, 14 U/mL; MMP-3, 17.0 ng/mL; PR3-ANCA, < 1.0 U/mL; MPO-ANCA, < 1.0 U/mL; sIL-2R, 445 U/mL; and T-SPOT, negative. Abdominal contrast-enhanced CT showed slight thickening of the small intestinal wall (Fig. 1). First, we performed a patency capsule examination to investigate the feasibility of a small bowel capsule endoscope. The patency capsule was not excreted, and it was confirmed by abdominal X-ray (Fig. 2). Abdominal contrast-enhanced CT revealed a contrast stagnation of the patency capsule in the proximal part of the ileum and mild dilatation of the proximal part (Fig. 3). A barium small bowel enema showed circumferential stenosis at the same site (Fig. 4). First, a transanal balloon endoscopy showed no abnormal findings, such as ulcerative lesions or stenosis, in the ileum. We injected carbon ink into the deepest part of the ileum. Subsequently, we performed an oral balloon endoscopy. We inserted the endoscope deep into the jejunum, and several shallow ulcerative lesions were noted (Fig. 5); however, we could not identify the inked part or stenotic lesions. A biopsy of the ulcerative lesion showed densely proliferating lymphocytes in the lamina propria and a marked reduction in gland ducts (Fig. 6). Immunohistochemistry for CD20 showed positive results with atypical lymphocytes. Based on these findings, we diagnosed B-cell LPD. Based on a history of oral administration of MTX, MTX-LPD was suspected. We discontinued MTX and followed up with the patient for two weeks. Despite the MTX withdrawal, her symptoms did not improve, and, therefore, surgery was required. During operation, an endoscope was inserted through the inked part of the small intestine to the oral and anal sides. We observed a semi-circumscribed ulcer and mild stenosis at 3 cm of the anal side from the inked part. However, we observed no other ulcerative lesions in the jejunum and ileum. After intraoperative endoscopic observation, we performed a partial resection of the small intestine, including the same site, with no adverse event. Histopathology of the resected specimen revealed atypical lymphoid cells immunostaining negative for CD3 and positive for CD20, consistent with follicular lymphoma (Fig. 7). The test result for Epstein-Barr virus (EBV)-encoded small RNA (EBER) was negative in the histological sample of surgical specimens. After the surgery, we found no evidence of lymphoma on fluorodeoxyglucose-positron emission tomography and bone marrow puncture. Therefore, we finally diagnosed follicular lymphoma localized in the small intestine with MTX-LPD. After the operation, her symptom improved, and we followed up without treatment. We have been following up with the patient on an outpatient basis for four years; no recurrence or complications have been reported.

## Discussion and conclusions

Although MTX is a first-line therapy for RA, it is a major cause of LPDs. The pathogenesis of MTX-LPD is incompletely understood; however, some studies have suggested that the “hyper-immune” state of RA and the immunosuppressive state associated with MTX might contribute to MTX-LPD development [7]. Although some reports conclude that lymphoma is not associated with MTX [8, 9], most reports showed that patients treated with MTX have an increased tendency of developing lymphoma. Most cases of MTX-LPD are patients with RA. The age of onset is wide, the median age is 67 years, and the male: female ratio is 1: 2. The mean duration of MTX treatment and mean cumulative dose of MTX-LPD development were 54 months (range, 2–131 months) and 940 mg (range, 24–4785 mg), respectively [10]. Over the past 10 years of treatment, our patient had received a cumulative dose of MTX over 3,000 mg, which is within the range specified in previous reports. In general, malignant lymphoma frequently occurs in lymph nodes, whereas MTX-LPD occurs in lymph node lesions and extranodal lesions. Extranodal lesions frequently occur in the digestive tract, skin, lungs, and soft tissues [9, 10]. Although the sub-classification of MTX-LPD has not been established, diffuse large B-cell lymphoma is the most common subtype of MTX-LPD. Other types of lymphoma include B-cell lymphoma, mucosa-associated lymphoid tissue lymphoma, Burkitt’s lymphoma, or follicular lymphoma, and sometimes even peripheral T-cell lymphoma or Hodgkin’s lymphoma [11]. Follicular lymphoma, as in this case, is extremely rare, with an incidence of approximately 3 % [12]. There are no markers of diagnostic tools for the early detection of MTX-LPD. Almost 50 % of patients with MTX-LPD show remission after the withdrawal of MTX [13], indicating the discontinuation of MTX as the first-line treatment. The patient was followed up two weeks after MTX discontinuation. Subsequent recurrence of MTX-LPD has been reported in 18–45 % of patients, and chemotherapy is indicated in cases of recurrence or those not reaching remission after three months of discontinuation. Chemotherapy was administered according to the histological type. EBV is an oncogenic virus involved in lymphomagenesis, such as Hodgkin’s lymphoma, Burkitt’s lymphoma, gastric carcinoma, and nasopharyngeal carcinoma [14]. Immunodeficiency is considered the basis for the onset of malignant lymphomas through the activation of EBV. Hoshida et al. [10] reported that the prevalence of EBV in RA patients with LPD was significantly higher than that in sporadic LPD (27.6 % vs. 9.9 %). In addition, 93.0 % of RA patients with LPD were serologically positive for EBV [15]. However, the test result for EBV was negative in this case. Miyazaki et al. [4] reported that the complete remission rate of MTX-LPD was under 30 % on withdrawal of MTX; however, the complete remission rate was as high as 60 % in the EBV-positive cases. In our case, intraoperative endoscopy showed no shallow ulcerative lesions of the jejunum; therefore, the withdrawal of MTX improved MTX-LPD instead of influencing the EBV negativity. However, the semi-circumferential ulcerative lesion and mild stenosis were unaffected. In this case, local resection of the small intestine and withdrawal of MTX resulted in complete remission. This is the first case report of follicular lymphoma in the small intestine with EBER negativity. However, there is a limitation that previous reports did not discuss EBER; therefore, the exact incidence of EBER negativity is not clarified.

In conclusion, we experienced the first case of MTX-LPD that was presumed to be follicular lymphoma. MTX-LPD can manifest in any pathological state and may occur in any extranodal organ. We should be aware of MTX-LPD when MTX is administered in RA patients. If intestinal symptoms occur during MTX administration, it is important to directly observe by endoscopy and perform histological examination.

## Data Availability

Data sharing is not applicable to this article as no datasets were generated or analyzed in this study.

## Abbreviations

MTX: Methotrexate
MTX-LPD: Methotrexate-associated lymphoproliferative disorder
CT: Computed tomography
RA: Rheumatoid arthritis
EBV: Epstein-Barr virus.
EBER: Epstein-Barr virus-encoded small RNA.

## References

[1] Smolen JS, Landewé R, Bijlsma J, Burmester G, Chatzidionysiou K, Dougados M (2017). EULAR recommendations for the management of rheumatoid arthritis with synthetic and biological disease-modifying antirheumatic drugs: 2016 update. Ann Rheum Dis.

[2] Gaulard P, Swerdlow SH, Harris NL, et al. Other iatrogenic immunodeficiency associated lymphoproliferative disorders. In: WHO Classification of Tumors of the Haematopoietics and Lymphoid Tissues. 4th ed. Lyon: International Agency for Research on Cancer; 2008. pp. 350–1.

[3] Tokuhira M, Tamaru JI, Kizaki M (2019). Clinical management for other iatrogenic immunodeficiency-associated lymphoproliferative disorders. J Clin Exp Hematop.

[4] Miyazaki T, Fujimaki K, Shirasugi Y, Yoshiba F, Ohsaka M, Miyazaki K (2007). Remission of lymphoma after withdrawal of methotrexate in rheumatoid arthritis: relationship with type of latent Epstein-Barr virus infection. Am J Hematol.

[5] Ishiguro K, Hayashi T, Aoki Y, Murakami R, Ikeda H, Ishida T (2016). Other iatrogenic immunodeficiency-associated lymphoproliferative disorder presenting as primary bone lymphoma in a patient with rheumatoid arthritis. Intern Med.

[6] Ichikawa A, Arakawa F, Kiyasu J, Sato K, Miyoshi H, Niino D (2013). Methotrexate/iatorogenic lymphoproliferative disorders in rheumatoid arthritis: histology, Epstein-Barr virus, and clonality are important predictors of disease progression and regression. Eur J Haematol.

[7] Toyonaga H, Fukushima M, Shimeno N, Inokuma T (2019). Methotrexate-associated lymphoproliferative disorder in the stomach and duodenum: a case report. BMC Gastroenterol.

[8] Wolfe F, Michaud K (2004). Lymphoma in rheumatoid arthritis: the effect of methotrexate and anti-tumor necrosis factor therapy in 18,572 patients. Arthritis Rheum.

[9] Mariette X, Cazals-Hatem D, Warszawki J, Liote F, Balandraud N, Sibilia J (2002). Lymphomas in rheumatoid arthritis patients treated with methotrexate: a 3-year prospective study in France. Blood.

[10] Hoshida Y, Xu JX, Fujita S, Nakamichi I, Ikeda JI, Tomita Y (2007). Lymphoproliferative disorders in rheumatoid arthritis: clinicopathological analysis of 76 cases in relation to methotrexate medication. J Rheumatol.

[11] Hasserjian RP, Chen S, Perkins SL, de Leval L, Kinney MC, Barry TS (2009). Immunomodulator agent-related lymphoproliferative disorders. Mod Pathol.

[12] Ichikawa A, Arakawa F, Kiyasu J, Sato K, Miyoshi H, Niino D (2013). Methotrexate/iatrogenic lymphoproliferative disorders in rheumatoid arthritis: histology, Epstein-Barr virus, and clonality are important predictors of disease progression and regression. Eur J Haematol.

[13] Saito S, Takeuchi T (2019). Immune response in LPD during methotrexate administration (MTX-LPD) in rheumatoid arthritis patients. J Clin Exp Hematop.

[14] Niedobitek G (2000). Epstein–Barr virus infection in the pathogenesis of nasopharyngeal carcinoma. Mol Pathol.

[15] Nalesnik MA, Jaffe R, Starzl TE, Demetris AJ, Porter K, Burnham JA, Makowka L, Ho M, Locker J (1988). The pathology of posttransplant lymphoproliferative disorders occurring in the setting of cyclosporine A-prednisone immunosuppression. Am J Pathol.

